# Safety & efficacy of thulium laser endoscopic en bloc resection versus conventional trans urethral resection of bladder tumors, for treatment of non muscle invasive bladder tumors: A prospective randomized trial

**DOI:** 10.1080/20905998.2025.2501888

**Published:** 2025-05-11

**Authors:** Mahmoud Abd El Hamid, Ahmed Abd Allah, Ahmed Assem, Mohamed Abd El Wahed, Hossam Hassan, Amr Lotfi, Ayman Kassem

**Affiliations:** Urology Department, Cairo University, Cairo, Egypt

**Keywords:** Thulium, superficial, TCC, non-invasive

## Abstract

**Introduction:**

Non-muscle invasive bladder cancers (NMIBCs) are managed with endoscopic resection and intravesical therapy. Lasers, particularly thulium and holmium, are now central to bladder tumor management. This study assesses the safety and efficacy of thulium laser enucleation of bladder tumors (Tm-LRBT).

**Patients & Methods:**

A prospective randomized clinical study was conducted from June 2022 to December 2023, involving 100 patients. Group A was treated with TmLRBT and Group B with conventional monopolar TURBT. Informed consent was obtained from all participants. Inclusion criteria were NMIBC, tumor size < 5 cm, and fewer than three tumors. Exclusion criteria included tumors > 5 cm, more than three tumors, invasive/upper tract cancers, hydronephrosis, metastases, and history of TURBT.

**Results:**

The mean tumor diameter in Group A was 2.3 ± 0.74 cm. The mean operative time was 45.4 ± 13.48 minutes. Re-resection within 90 days was negative for bladder cancer in all Group A patients; three patients in Group B had persistent disease. Seven Group B cases lacked muscle in the specimen compared to three in Group A. Significant intraoperative bleeding occurred in four cases in Group A and five in Group B. Tumor recurrence was 42% in Group A and 44% in Group B at 12-month follow-up.

**Conclusion:**

TmLRBT is a potential alternative to TURBT, providing accurate reporting of neoplastic depth invasion. It is advantageous, particularly for tumors in the lateral wall, bladder dome, or perimeatal zone.

## Introduction

Bladder cancer is one of the most common cancers among men in Europe and America, ranking fourth after prostate, lung, and colon cancers. However, its high recurrence rates make it potentially the most prevalent. In Egypt, bladder cancer remains the most frequent malignant tumor among Egyptian males [1,2].

Disease recurrence occurs in up to 80% of cases with non-muscle invasive bladder cancer (NMIBC) and is a major issue for Ta NMIBC patients. Disease progression occurs in up to 30% of cases and poses a significant threat to those with T1 or carcinoma in situ (CIS). Generally, NMIBCs are managed with endoscopic resection and risk-based intravesical therapy (bladder instillation), while muscle-invasive bladder cancers (MIBCs) require radical cystectomy with or without chemotherapy [3–6].

The introduction of en bloc resection of urinary bladder tumors has brought lasers into focus for bladder tumor management. The most commonly used are thulium and holmium lasers Various research groups have reported promising results for laser en bloc resection of bladder tumors, including pathology assessment and recurrence rates. Additionally, many studies have shown better patient safety outcomes with laser en bloc resection, such as less bleeding, no obturator jerk leading to urinary bladder trauma, and decreased perioperative complications [7,8].

This study aims to assess the safety and efficacy of thulium laser en bloc resection of bladder tumor (Tm-ERBT) in comparison to conventional monopolar TURBT (transurethral resection of bladder tumors).

## Patients and methods

This prospective randomized clinical comparative study was conducted in the uro-oncology unit of the Urology Department, Cairo University between June 2022 and December 2023, involving 100 patients.

### Sample size calculation

The primary outcome was the incidence of detrusor muscle presence in patients with non-muscle invasive bladder cancer treated with TmERBT versus conventional TURBT. Sample size calculation was based on comparing two proportions from independent samples using the Proportion: inequality, two independent groups (Fisher’s exact test), with an α-error level of 0.05, power of 95%, and an intervention group ratio of 1. As previously published [9], the incidence of detrusor muscle presence was 58.6% for Thulium laser and 91.6% for conventional resection. The minimum optimal sample size was 46 participants per group, calculated using G Power for Sample Size Calculations software, version 3.1.9.7. We added 10% as attrition rate of patients who may miss the follow-up, so our sample size was 50 participants per group.

**Figure 1.**
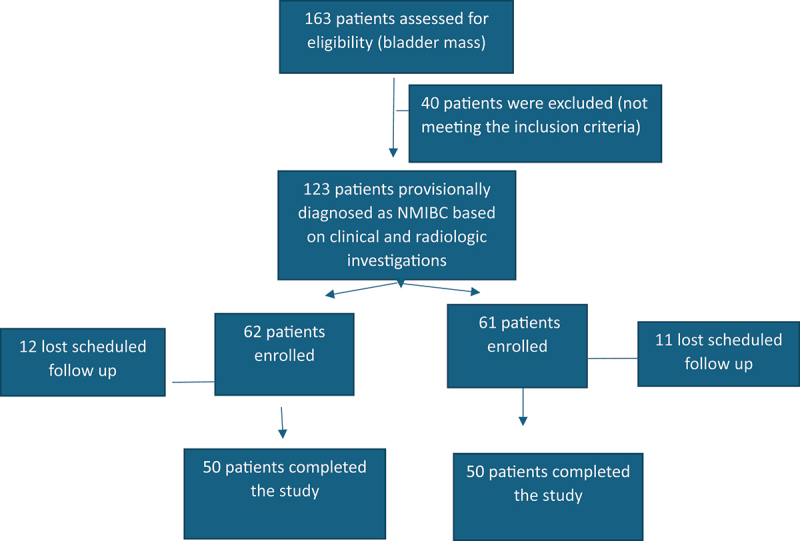
CONSORT flow diagram for cases allocation in the study.

Out of 163 patients admitted with bladder mass, & diagnosed by CT urography or MRI, 123 met the inclusion criteria, and were diagnosed by imaging (U/S, CT scans, or MRI), and Cystoscopy. They were randomized into two groups, group A to undergo TmLRBT, & group B to undergo conventional TURBT, randomization was computer based using random number generator plus version 2.4.8. Twelve patients in group A lost scheduled follow-up, leaving 50 patients enrolled in the study. Eleven patients in group B lost scheduled follow-up, also leaving 50 patients enrolled in the study. Which met our estimated optimum sample size.

an informed consent was signed by the patient after being informed about the procedure, risks, and potential benefits. (Figure 1) The study was approved by the ethical committee (Faculty of medicine, Cairo University), and was registered under IRB No. MD-183-2022.

### Inclusion criteria

Patients with NMIBC, tumor size less than 5 cm, and fewer than three tumors. **Exclusion Criteria**: Those not meeting inclusion criteria, MIBC, upper tract urothelial carcinoma, hydronephrosis, metastases on imaging, and past history of TURBT.

Demographic data were collected and included age, sex, medical history, mass localization, and size. A detailed history was obtained, focusing on presenting complaints, symptom duration, and comorbid conditions. Pre-operative labs included CBC, coagulation profile, kidney functions, urinalysis, culture and sensitivity.

The size of the mass measured preoperatively by CT scan or MRI, and intraoperatively by the surgeon using the current loop as a measure (1 cm between the tip of the loop and the lens) in conventional TURBT, and in TmERBT group using a graded ureteric catheter passed through the working channel to measure the tumor size.

### Surgical technique

The procedure started by a thorough cystoscopic examination. TmERBT was performed in lithotomy position under spinal anesthesia, using 0.9% sodium chloride for continuous irrigation. TmERBT was conducted through a 550-μm optical laser fiber (RigiFibTM, LISA, Katlenburg, Germany) introduced into a 26F continuous-flow resectoscope (Karl Storz, GmbH, Tuttlingen, Germany). The fibre was connected to the Tm: YAG laser (RevolixR, LISA, Katlenburg, Germany) in continuous-wave mode at 30 W, with parameters set to 1.5 J energy, 20 hz pulses, and 30 W power.

**Figure 2.**
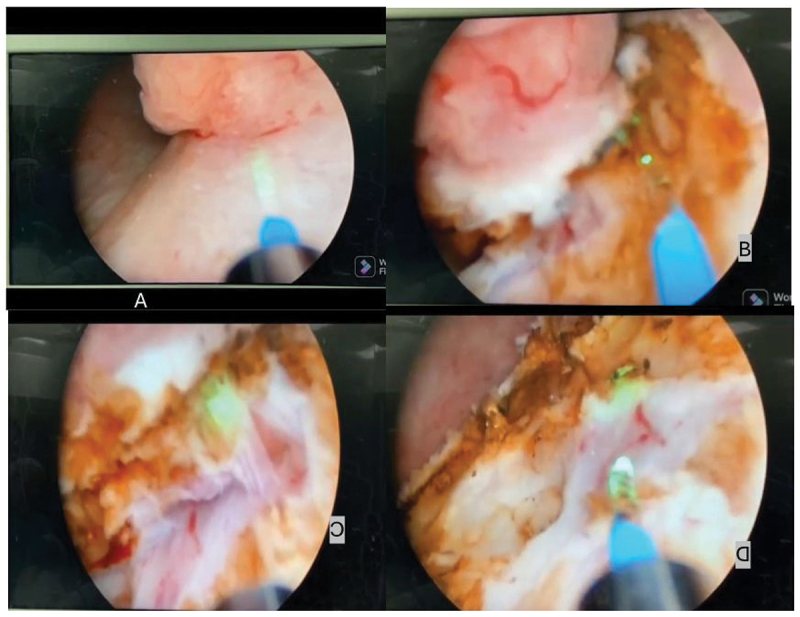
Stages of TmLRBT (a. incision of bladder mucosa, b.Identification of enucleation plane at the loose space between the muscular layer and the connective tissue layer,c. separation of detrusor muscle fibres from underlying base, d. hemostasis of base of resection after separation of the tumor).

For small villous tumors with distinct stalks, a 2–5 mm circumferential incision was made in the bladder mucosa. For non-pedunculated tumors, a slight forward pushing movement using the resectoscope tip (mechanical dissection) was applied (Figure 2a). After cutting the mucosa, the resectoscope sheath tip and thulium laser vaporization power was used to push the tumor base until the submucosa was exposed, identifying the fibrous connective tissue between the mucosal layer and detrusor muscle (Figure 2b). Through layer-by-layer resection, detrusor muscle fibers were removed, followed by blunt dissection along the loose space between the muscular and connective tissue layers. Muscle fibers were cut from the tumor base if connected, achieving endoscopic resection (Figure 2c). Saline irrigation was provided throughout, with the fiber tip contacting the tumor tissue accurately to avoid thermal damage. For tumors on the anterior bladder wall, gentle and steady suprapubic pressure facilitated enucleation using the resectoscope, which was rotated around the tumor. For tumors at the bladder dome, minimal mechanical dissection was performed in a semi-filled bladder, relying primarily on laser resection after reaching the desired plane to avoid intraperitoneal perforation.

To prevent tumor cell spillage, tumors smaller than 2.5 cm were grasped and enucleated between the resectoscope loop and sheath, and then extracted as a single unit through the urethra. For tumors 2.5 cm or larger, a cross-sectional or longitudinal incision was made to divide the tumor into two pieces while it remained attached at the edge, resembling a ‘moving leaf attached to its branch.’ These pieces were then removed (Figure 3a).

**Figure 3a.**
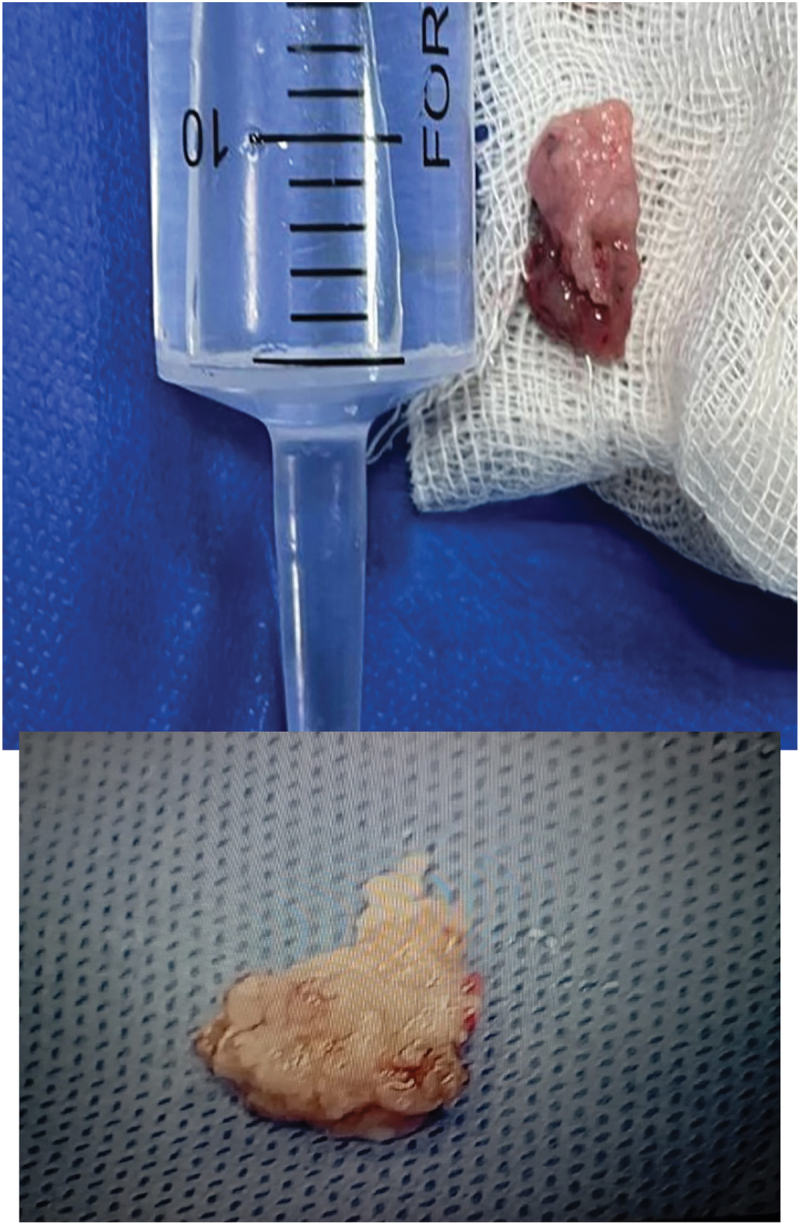
A: specimen of thulium laser enbloc resection.

At the end of the resection, coagulation of the tumor base using the thulium laser in pulsed-wave mode at 20 W. (Figure 2d) Continuous bladder irrigation (CBI) was initiated if necessary, Specimens sent for histopathological assessment. Postoperative bladder irrigation stopped if clear urine. Patients were informed about possible late complications. Postoperative intravesical chemotherapy was considered according to EAU guidelines if no contraindications.

Monopolar TURBT was performed with settings of 100–110 W for cutting and 60 W for coagulation. Tumors smaller than 10 mm were resected in one piece. While Larger tumors were resected in fractions, including the exophytic part, underlying bladder wall including detrusor muscle, and resection area edges (Figure 3b).

**Figure 3b.**
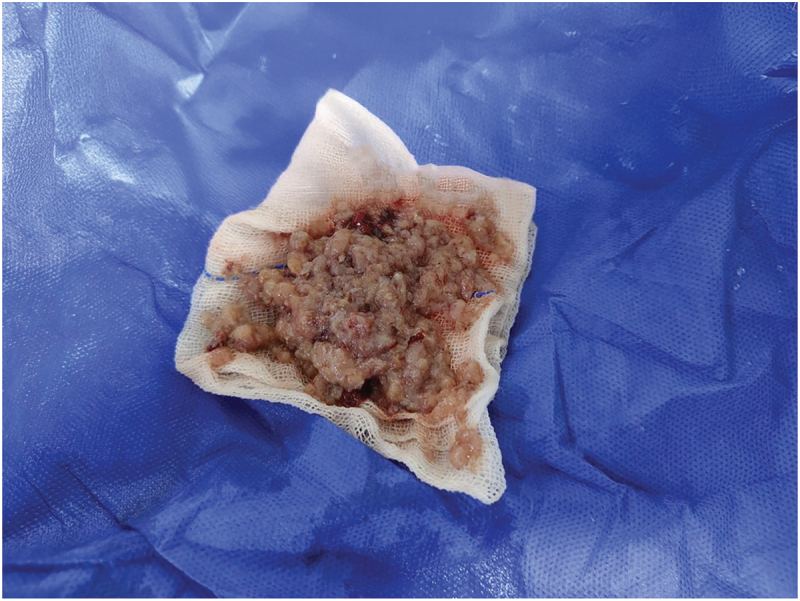
B: specimen of conventional TURBT.

Both TmERBT and TURBT were performed by two senior urologists in the uro-oncology unit, Urology Department, Cairo University, with over 100 cases of experience in endo-urology and 50 cases in laser resection of bladder tumors.

### Data collection

Perioperative data included cystoscopy report, operative time, number, site, and size of lesions, complications (perforation, obturator reflex, & bleeding), irrigation duration if needed, urethral catheterization duration, hospital stay, and adjuvant intravesical therapy.

Perforation was assessed clinically, (based on abdominal examination, vital signs instability, significant difference in volume of irrigation fluid drained from the endoscopic sheath), & radiologically by performing on table cystography for suspected cases.

**Restaging TURBT** was performed in each group 2–6 weeks after the first cystoscopy. Follow-up cystoscopies were conducted according to EAU guidelines risk stratification [10].

### Adjuvant chemotherapy

Immediate intra-vesical chemotherapy (40 mg of mitomycin) was given to all patients except in cases of perforation, within the first two hours after surgery. Based on EAU risk stratification, all high-risk patients received BCG maintenance adjuvant therapy, while intermediate-risk patients received adjuvant intravesical chemotherapy, and low-risk patients received no adjuvant therapy.

### Pathology

Stage and grade of the tumor, presence of muscular layer in the specimen, and specimen quality.

### Primary outcome

Incidence of detrusor muscle presence in patients with non-muscle invasive bladder cancer treated with TmLRBT versus conventional TURBT.

### Secondary outcome

Perforation rate of thulium laser resection of bladder tumors in various bladder walls. Perforation was assessed by clinical suspicion & conventional cystography performed to suspected cases.

Our follow-up protocol, entailed patients classification to: Low risk (single, low-grade Ta, less than 3 cm with no associated CIS) who were scheduled for cystoscopy at 3 and 12 months. Intermediate and high risk, who were scheduled for cystoscopy at 3, 6, 9, and 12 months and received adjuvant therapy, all patients had a detailed history taking, urine analysis, examination, & urine cytology done before each scheduled cystoscopy.

### Statistical analysis

Data were coded and entered using the statistical package for the Social Sciences (SPSS) version 28 (IBM Corp., Armonk, NY, USA). Data was summarized using mean, standard deviation, minimum and maximum for quantitative variables and frequencies (number of cases) and relative frequencies (percentages) for categorical variables. Comparisons between groups were done using unpaired t test in normally distributed quantitative variables while non-parametric MannWhitney test was used for non-normally distributed quantitative variables [11]. For comparing categorical data, Chi square (2) test was performed. Exact test was used instead when the expected frequency is less than 5 [12]. P-values less than 0.05 were considered as statistically significant.

## Results

This is a prospective randomized comparative trial including 100 patients with NMIBC, divided into two groups, we started our study with 163 patients admitted to our uro-oncology unit with bladder masses, 40 patients didn’t meet the inclusion criteria, 123 patients were randomized into two groups A & B. Group A to receive TmLRBT and group B to receive TURBT. Randomization was computer based using random number generator plus version 2.4.8. Diagnostic cystoscopy was done to all patients. Twelve patients in group A lost scheduled follow-up, leaving 50 patients enrolled in the study. Eleven patients in group B lost scheduled follow-up, also leaving 50 patients enrolled in the study.

There were no significant statistical differences between the groups regarding demographic parameters (*p* value > 0.05), with the mean age in group A being 58.94 ± 10.835 years (range 30–81) and in group B being 59.9 ± 7.5 years (range 44–75). Additionally, 44% of group A & 48% of group B were smokers, as shown in Table 1.

**Table 1.** Sociodemographic features and comorbidities of the study group.

		Laser en bloc Resection (A) (*n* = 50)	Conventional TURBT (B) (*n* = 50)	*p* value
Age (/years) Mean ±SD Range		58.94 ± 10.835 30–81	59.9 ± 7.5 44–75	0.601
Sex Number (%)	Male	43 (86%)	42 (84%)	0.779
	Female	7 (14%)	8 (16%)	
Past medical history Number (%)	DM	11 (22%)	7 (14%)	0.298
	HTN	11 (22%)	6 (12%)	0.143
	IHD	4 (8%)	4 (8%)	0.643
	Others	6 (12%)	5 (10%)	0.415
Smoking (Number (%)		22 (44%)	24 (48%)	0.421

Tumour characteristics such as count, size, and sites of masses didn’t show a statistically significant difference between both groups (*p* value > 0.05). The mean number of masses was 1.64 ± 0.74 in group A and 1.56 ± 0.67 in group B. Mean size of masses was 2.3 ± 0.73 cm (range 1.3–5) in group A and 2.2 ± 0.66 cm (range 1–4.5) in group B, with no difference in the distribution of bladder masses across the bladder walls in both groups, as shown in Table 2.

**Table 2.** Tumor characteristics of the study groups.

		Group (A) (*n* = 50)	Group (B) (*n* = 50)	*p* value
Number of masses	Mean ± SD	1.64 ± 0.74	1.56 ± 0.67	0.601
	Range	1–3	1–3	
Size of mass (in cm)	Mean ± SD	2.3 ± 0.73	2.2 ± 0.66	0.576
	Range	1.3–5.0	1.0–4.5	
Site Number (%)	Posterior wall	6 (12%)	12 (18%)	0.288
	Lateral wall	23 (46%)	26 (52%)	0.588
	Peri-meatal	8 (16%)	8 (16%)	0.243
	Bladder base	9 (18%)	8 (16%)	0.500
	Anterior wall	7 (14%)	8 (16%)	0.779
	Bladder dome	7 (14%)	10 (20%)	0.298
	Bladder neck	6 (12%)	3 (6%)	0.243
	Prostatic urethra	6 (12%)	1 (2%)	0.056

**The primary end point of this study** was the presence of detrusor muscle in initial resection specimen, detrusor muscle was not detected in 3 cases (6%) in group A and 7 cases (14%) in group B, with no significant statistical difference between both groups (*p* value = 0.213).

Perioperative parameters showed no statistically significant difference between the studied groups regarding bleeding, irrigation time, catheterization time, and hospital stay (Table 3).

**Table 3.** Comparison of perioperative data of the two groups.

			Group (A) (*n* = 50)	Group (B) (*n* = 50)	*p* value
Operative time (in minutes)	Mean ± SD		49.20 ± 15.16	42.3 ± 14.44	0.022
	Range		30.0–90.0	120.0–90.0	
Irrigation time	Mean ± SD		15 ± 5.55	19.5 ± 3	0.283
	Range		6–18	18–24	
Perforation (number (%)			0	5 (10%)	0.028
Bleeding (number (%)			2 (4%)	4 (8%)	0.678
Obturator jerk (number (%)			0 (0%)	8 (16%)	0.006
Catheter time (in days)		Mean ± SD	3.92 ± 2.25	4 ± 2.28	0.908
		Range	3.0–14.0	3.0–14.0	
Hospital stay (in days)		Mean ± SD	2.6 ± 0.24	2.3 ± 1.18	0.172
		Range	2.0–3.0	2.0–10	

**The Secondary end point** of the study was the perforation rate, in fact there was a statistically significant difference in perforation rate among the two groups, as we didn’t encounter perforation in group A, while, unfortunately 5 cases were perforated in group B (*p* value < 0.05). In addition, there was a statistically significant difference in obturator jerk incidence among both groups, with no case in group A, and 8 cases in group B. No case in group A was converted to conventional TURBT, which proves the feasibility of TmERBT for any mass up to 5 cm in diameter across different bladder walls. However, there was a statistically significant difference in operative time, which was longer in group A (Table 3). No patients required blood transfusion or ICU admission.

Histopathological assessment of the retrieved specimens, showed that muscularis mucosa was detected in all pathology specimens in both groups, non of the cases had lymphovascular invasion, or any associated carcinoma in situ, nor other differentiations in both groups. Group A showed that 62% were T1 high grade, 12% were Ta high grade, and 26% were Ta low grade. In group B, 60% were T1 high grade, 20% were Ta high grade, and 20% were Ta low grade, with no significant difference between both groups (Table 4).

**Table 4.** Comparison between the two groups regarding post resection staging.

		Group (A) (*n* = 50)	Group (B) (*n* = 50)	*p* value
Muscle presence (Number %)		47 (94%)	43 (86%)	0.213
Staging (Number %)	Ta	19 (38%)	20 (40%)	0.495
	T1	31 (62%)	30 (60%)	
	Low grade	13 (26%)	10 (20%)	
	High grade	37 (74%)	40 (80%)	
	Ta low grade	13 (26%)	10 (20%)	
	Ta high grade	6 (12%)	10 (20%)	
	T1 high grade	31 (62%)	30 (60%)	

**Re staging TURBT** was conducted after 2–6 weeks (21 ± 3 days) 36 patients in group A and 40 patients in group B (in cases of T1, high-grade tumour, or absent muscle in the specimen of primary cystoscopy). Aiming for resection of any residual lesion and to take a biopsy from the base of previous resection. No visible residual tumour was detected in group A but biopsy from resection base revealed MIBC in two cases, while 3 cases of residual tumour were found in group B. Five cases were upstaged to muscle-invasive bladder cancer (2 in group A and 3 in group B).

Based on the EUA guidelines risk stratification of NMIBC, 74% of group A were high risk compared to 80% of group B, 16% of group A were intermediate risk compared to 8% of group B, and 10% of group A were low risk compared to 12% of group B. All high-risk patients received BCG maintenance adjuvant therapy, while intermediate-risk patients received adjuvant intravesical chemotherapy, and low-risk patients received no adjuvant therapy.

During the 12-month follow-up, all patients adhered to the surveillance regimen as advised by the EUA guidelines (every 3 months for high and intermediate risk, and at 3 and 12 months for low risk). Tumor recurrence was observed in 21 cases (42%) in Group A (3 at 3-month, 3 at 6-month, 8 at 9-month, & 7 at 12-month follow-up) and 22 cases (44%) in Group B (4 at 3-month, 6 at 6-month, 8 at 9-month, & 4 at 12-month follow-up) (Table 5).

**Table 5.** Comparison between both groups regarding tumor recurrence.

		Group (A) (*n* = 50)	Group (B) (*n* = 50)	*p* value
Recurrence (Number %)		21 (42 %)	22 (44 %)	0.84
Time of recurrence (Number %)	At 3 months	3 (14.3%)	4 (18.2%)	0.64
	At 6 months	3 (14.3%)	6 (27.3%)	
	At 9 months	8 (38.10%)	8 (36.4%)	
	At 12 months	7 (33.3%)	4 (18.2%)	

Logistic regression was done to detect independent predictors of recurrence and results showed that in univariate analysis, tumor stage (T1), grade (high grade) number (multiple), and size of the mass are significant predictors of recurrence while the treatment modality did not affect the recurrence rate. Yet in multivariate analysis, high grade, multiple masses, and big size of mass act as independent predictors of recurrence with Odds ratio > 1 meaning that they increased risk of recurrence (Tables 6 and 7).

**Table 6.** Univariate logistic regression for prediction of recurrence in both groups.

		*P* value	OR	95% C.I.	
				Lower	Upper
Recurrence	Modality (Laser)	0.840	0.922	0.417	2.035
	Stage (T1)	0.019	2.809	1.189	6.635
	Grade (high)	0.008	4.875	1.517	15.662
	number of masses (multiple)	<0.001	8.233	2.748	24.671
	size of mass	0.005	1.842	1.202	2.823

**Table 7.** Multivariate logistic regression for prediction of recurrence.

		*P* value	OR	95% C.I.	
				Lower	Upper
Recurrence	Stage (T1)	0.964	1.030	0.286	3.712
	Grade (high)	0.023	7.780	1.319	45.908
	number of masses (multiple)	<0.001	17.966	4.610	70.012
	size of mass	0.001	2.676	1.505	4.758

## Discussion

Transurethral resection of bladder tumor (TURBT) is the cornerstone of treatment for non-muscle invasive bladder cancer (NMIBC). Conventional TURBT involves resecting the bladder tumor into smaller, removable fragments with an adequate muscle base to ensure proper staging and cancer control. Despite being the standard for a long time, conventional TURBT carries risks of incomplete resection and tumor recurrence [13].

The primary goals of en bloc resection of bladder tumors (ERBT) are to achieve adequate cancer control, improve staging quality by obtaining detrusor muscle in the specimen, and minimize postoperative complications. Postoperative hematuria, with or without clot retention, is the most common complication following TURBT, while bladder perforation remains the most significant complication and can be associated with unfavourable oncological outcomes [14].

Monopolar and bipolar TURBT have traditionally been used for resection and enucleation of bladder tumors. Despite their efficacy, these procedures can be associated with significant complications such as bladder perforation and resection failure. Additionally, the current flow during resection of lateral bladder wall tumors can trigger the obturator nerve reflex (ONR) in about 12–25% of patients, leading to a perforation rate of 2–10%. Techniques such as using bipolar electrodes and obturator nerve block have been suggested to avoid ONR. However, the efficacy of bipolar electrodes in preventing ONR is still debated. Successful obturator nerve blockage may require ultrasound or nerve stimulator assistance, making the procedure time-consuming and operator-dependent [15–18].

Thulium laser was first introduced in urologic practice in 2005 and has since been used in various urological fields, including stone lithotripsy, prostatic adenoma resection or vaporization, and recently, ERBT. Thulium laser technology offers several advantages for ERBT, including a continuous wave pattern and shallow penetration depth (250 μm), providing a sharp incision with clear vision and a lower risk of bladder perforation. Additionally, the laser’s non-electric energy does not trigger the obturator nerve reflex, making the procedure safe for lateral wall bladder tumors and patients with cardiac pacemakers [19].

In this study, 100 patients with bladder cancer were enrolled, after meeting the inclusion criteria, and completing the scheduled follow-up for 12 months, they were randomly divided into two groups, and underwent either thulium laser resection (group A) or monopolar resection (group B).

The presence of detrusor muscle is essential for specimen adequacy while avoiding perforation, which is the primary endpoint of our study. Having the fact that the penetration depth of thulium laser is 0.2 mm, compared to 1 mm for monopolar current, and that this higher penetration depth for monopolar current increases the risk of bladder perforation and the potential for extravesical tumor cell seeding, impacting oncological outcomes, renders TmERBT feasible and safe for resection of NMIBC [19]. Following conventional TURBT, detrusor muscle is reportedly included in approximately 83.3% of specimens [20]. These results align with our study findings for the TmLRBT group (group A vs. group B: 94% vs. 86%), with no significant statistical difference between the groups regarding detrusor muscle acquisition (*p* = 0.213). Additionally, bladder perforation did not occur in group A (0 cases) but was observed in five cases in group B, with a significant statistical difference (*p* = 0.028). Consistent with this study, many authors have reported a high percentage of detrusor muscle inclusion during Tm-LRBT, up to 100% of specimens [13,21,22].

Adding to this, the low perforation rate which we encountered in group A, in comparison to 5 cases in group B, (*p* < 0.05), & the statistically significant difference in obturator jerk incidence among both groups, with no case in group A, and 8 cases in group B. In addition, we didn’t convert any case in group A to conventional TURBT, proves the safety of TmERBT, which was the same as the study done by Enikeev, Dmitry, et al, who didn’t encounter any case of obturator jerk or perforation in their cohort of patients who were treated with TmERBT [9].

Some authors have stated that certain tumor locations, such as domal, anterior wall, and high posterior wall lesions, may be challenging to resect due to technical considerations [13]. However, in our study, all tumors at various bladder walls were accessible for complete resection, with zero resection failures in both groups and no higher incidence of tumor location-related complications. Similarly, Migliari et al. found Tm-ERBT safe and feasible for tumor resection at various bladder walls [13].

The need for re-staging bladder cancer after complete tumor resection, particularly in T1 high-grade tumors, is crucial based on the possibility of upstaging the tumor status during re-TURBT, which can occur in about 33–53% of high-grade tumors and 40% of Ta high grade tumors in initial TURBT specimens. Re-TURBT, though highly indicated for T1 high-grade tumors without included detrusor muscle, is associated with a higher incidence of complications than initial TURBT [23–26]. In this study, re staging TURBT was conducted after 2–6 weeks in 36 patients in group A and 40 patients in group B (in cases of T1, high-grade tumour, or absent muscle in the specimen of primary cystoscopy). No visible residual tumour was detected in group A, while 3 cases of residual tumour were found in group B. Five cases were upstaged to muscle-invasive bladder cancer (2 in group A and 3 in group B).

For tumor recurrence, no statistically significant difference was found between the studied groups (*p* = 0.081). Which coincides with a meta-analysis by Long et al., who found no statistically significant difference (*p* = 0.052). In fact, due to the small number of included studies, further assessment of the recurrence rate with larger patient cohorts is needed.

The en bloc technique of Tm-ERBT allows for complete tumour removal with a low risk of dissemination. For large tumours requiring longitudinal incision, the coagulation layer beneath the vaporized tissue part minimizes the possibility of tumour cell seeding. Whether thulium ERBT can offer superior cancer control compared to TURBT requires further studies. The main limitations of this study were the inability to extract large tumours in one piece, necessitating bisection, and the relatively small number of participants. Additionally, the absence of bipolar surgery was a limitation, though bipolar TURBT is not necessarily safer than monopolar TURBT [27]. Photodynamic diagnosis (PDD) and narrow-band imaging (NBI) were not used.

## Conclusion

Compared to conventional TURBT, TmERBT showed superior safety with fewer intraoperative complications, less risk of postoperative bleeding, and non-inferior efficacy in cancer control. TmERBT requires longer operative time, yet, it is an effective option for NMIBC.
